# Editorial: The molecular regulation of microbial metabolism

**DOI:** 10.3389/fcell.2024.1458625

**Published:** 2024-07-16

**Authors:** Wenbin Yu, Yufei Zhang, Zhiwei Ouyang, Yayi Tu, Bin He

**Affiliations:** Jiangxi Key Laboratory of Natural Microbial Medicine Research, College of Life Sciences, Jiangxi Science and Technology Normal University, Nanchang, Jiangxi, China

**Keywords:** biomarker, signalling, secondary metabolism, metabolic switch, strain engineering

Microorganisms are integral to ecosystems, serving as key producers of natural products with broad implications for pharmaceuticals, agriculture, and industry (Jansson et al., 2023). Their metabolism drives the biodegradation of pollutants and the synthesis of essential compounds such as antibiotics and biofuels (Choi et al., 2023), influencing ecosystems ranging from soil microbiota to the human gut microbiome. This underscores the critical role of microorganisms in ecological balance and human health. Advancing our understanding of the molecular mechanisms underlying microbial metabolic regulation is crucial. Research has begun to unravel the complex interplay of enzymatic activities, regulatory networks, and adaptations, providing foundational knowledge that can be harnessed for innovative environmental and health applications (Liddicoat et al., 2024). The Research Topic titled “*The Molecular Regulation of Microbial Metabolism*” have illuminated key aspects of microbial metabolic regulation, shedding light on the intricate molecular mechanisms that govern metabolic pathways across various microbial species. Understanding enzymatic activities, regulatory networks, and metabolic adaptations in microorganisms has both advanced our basic knowledge of their physiology and spurred innovative applications in biotechnology and environmental science.

Whole genome sequencing and computational models have clarified how microorganisms optimize metabolic fluxes. The qORAC theory explains their dynamic gene expression adjustments for maximal metabolic efficiency, often involving post-translational modifications of transcription factors and metabolites. This concept builds on the traditional view of metabolic reprogramming as a key to organismal adaptation (Planqué et al., 2018). The concept of “metabolic reprogramming” has traditionally been central to our understanding of how organisms adapt to new conditions through changes in gene expression. However, as Rothman et al. emphasize, the pre-existing metabolic state and the metabolic flexibility of organism are equally pivotal. Understanding the metabolic properties of organism before an environmental shift is crucial for comprehending its survival, subsequent genetic adaptations, and resulting phenotypes. This perspective calls for a more holistic approach to studying metabolic adaptations, one that recognizes the contributions of both metabolic and genetic factors.

Traditionally, *Streptomyces* species are known to initiate antibiotic production under conditions of phosphate limitation, which coincide with low ATP levels. However, the deliberate introduction of ATP wasting as a strategy to enhance metabolic activity has been less explored in this genus. Apel et al. introduced the small synthetic protein DX, which has a high affinity for ATP and catalyzes its conversion to ADP, thereby simulating an ATP spilling effect. By cloning the gene encoding DX into the integrative vector pOSV10 and introducing it into *Streptomyces albogriseolus/viridodiastaticus*, the study creates a model strain, A37, which exhibits increased biomass production compared to the control strain A36.

Transcription factors play a key role in microbial metabolic pathways, and their regulation can affect the metabolic efficiency and adaptability of microorganisms (Yu et al., 2023). The study of the Lrp regulator protein in *Haloferax mediterranei* highlights its crucial role in nitrogen metabolism, illustrating the complex regulatory mechanisms that allow extremophiles to precisely adapt their metabolism to environmental changes through the intricate interplay between Lrp and its target gene promoters (Matarredona et al., 2024). Martin unveiled another layer of metabolic regulation, the study of calcium-responsive proteins and transcriptional factors in yeasts and fungi. Phosphate and calcium ions, crucial for growth, differentiation, and the production of bioactive secondary metabolites in filamentous fungi, are intricately linked through the master Pho4 transcriptional factor. This interaction, along with the role of TRPCa7-like channels in the expression of Pho89, underscores the significance of CrzA and Pho89 in the crosstalk between phosphate and calcium regulatory pathways. Furthermore, the discovery of acidocalcisomes in mycorrhiza and certain melanin-producing fungi, which exhibit characteristics akin to protozoan calcisomes, has opened new avenues for understanding the close interaction between orthophosphate, pyrophosphate, polyphosphate, and calcium ions within these organelles. However, the full spectrum of gene expression control remains to be fully explored.

Understanding the intricate interplay between microbial metabolism and host health responses represents a pivotal frontier in current research. Adegboro and Afolabi have comprehensively dissected the molecular mechanisms underlying ferroptosis, a distinct iron-mediated cell death pathway that requires diverse types of cellular metabolism. Their research focuses on how mitochondria modulate the susceptibility of malaria parasites to ferroptosis. They explore the intricate interplay among mitochondrial function, glutathione depletion, and the accumulation of toxic lipid reactive species, offering a novel perspective on inducing a ferroptosis-mediated antimalarial response by disrupting the redox balance in malaria parasites through mitochondrial targeting. In this context, the functional roles of ADP-ribosylation writers, readers, and erasers, as discussed by Li et al., added another dimension to our understanding of molecular regulation. ADP-ribosylation, a reversible post-translational modification (PTM), is intricately regulated by the dynamic interactions among its key players. This versatile PTM is pivotal in a multitude of physiological and pathological processes, and its dysregulation has been implicated in various diseases. The elucidation of the ADP-ribosylation cycle and the roles of its enzymes may not only facilitate the investigation of its functions but also contribute to the development of therapeutic strategies for associated diseases.

In conclusion, the molecular regulation of microbial metabolism is a sophisticated orchestration of genetic and metabolic factors that shape the adaptability and resilience of microorganisms. The studies here also highlight the critical importance of pre-existing metabolic states and the organism’s metabolic flexibility in the face of environmental perturbations. The exploration of ATP wasting in *Streptomyces* species, alongside the deep dive into transcription factors and ADP-ribosylation, showcase our growing ability to predict and the molecular regulation of microbial metabolism. However, the complexity of metabolic networks and the subtle interplay between genetic and environmental factors demand ongoing investigation. It becomes clear that a holistic approach is essential. This approach must integrate the strategic management of cellular stress, and the dynamic responses to environmental cues.
